# Simulating the impact of centralization of prostate cancer surgery services on travel burden and equity in the English National Health Service: A national population based model for health service re‐design

**DOI:** 10.1002/cam4.3073

**Published:** 2020-04-23

**Authors:** Ajay Aggarwal, Stéphanie A. van der Geest, Daniel Lewis, Jan van der Meulen, Marco Varkevisser

**Affiliations:** ^1^ Department of Cancer Epidemiology, Population and Global Health King’s College London London UK; ^2^ Department of Clinical Oncology Guy’s & St Thomas’ NHS Trust London UK; ^3^ Erasmus School of Health Policy and Management Erasmus University Rotterdam Rotterdam The Netherlands; ^4^ Department of Social and Environment Health Research London School of Hygiene and Tropical Medicine London UK; ^5^ Department of Health Services Research and Policy London School of Hygiene and Tropical Medicine London UK

**Keywords:** cancer surgery, centralization, equity, health service redesign, patient preference, travel time

## Abstract

**Introduction:**

There is limited evidence on the impact of centralization of cancer treatment services on patient travel burden and access to treatment. Using prostate cancer surgery as an example, this national study analysis aims to simulate the effect of different centralization scenarios on the number of center closures, patient travel times, and equity in access.

**Methods:**

We used patient‐level data on all men (n = 19,256) undergoing radical prostatectomy in the English National Health Service between January 1, 2010 and December 31, 2014, and considered three scenarios for centralization of prostate cancer surgery services A: procedure volume, B: availability of specialized services, and C: optimization of capacity. The probability of patients travelling to each of the remaining centers in the choice set was predicted using a conditional logit model, based on preferences revealed through actual hospital selections. Multivariable linear regression analysed the impact on travel time according to patient characteristics.

**Results:**

Scenarios A, B, and C resulted in the closure of 28, 24, and 37 of the 65 radical prostatectomy centers, respectively, affecting 3993 (21%), 5763 (30%), and 7896 (41%) of the men in the study. Despite similar numbers of center closures the expected average increase on travel time was very different for scenario B (+15 minutes) and A (+28 minutes). A distance minimization approach, assigning patients to their next nearest center, with patient preferences not considered, estimated a lower impact on travel burden in all scenarios. The additional travel burden on older, sicker, less affluent patients was evident, but where significant, the absolute difference was very small.

**Conclusion:**

The study provides an innovative simulation approach using national patient‐level datasets, patient preferences based on actual hospital selections, and personal characteristics to inform health service planning. With this approach, we demonstrated for prostate cancer surgery that three different centralization scenarios would lead to similar number of center closures but to different increases in patient travel time, whilst all having a minimal impact on equity.

## Graphical abstract

The study provides an innovative simulation approach using national patient‐level datasets, patient preferences based on actual hospital selections, and personal characteristics to inform health service planning. With this approach, we demonstrated for prostate cancer surgery that three different centralization scenarios would lead to similar numbers of center closures but to different increases in patient travel time, whilst all having a minimal impact on equity.

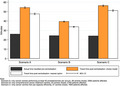

## 1 INTRODUCTION

The centralization of complex cancer surgery into high‐volume units is occurring in most high‐income countries as a consequence of a range of policies aiming to improve the quality and efficiency of cancer services.^1^, ^2^, ^3^ This has been in response to studies from predominantly Europe and the United States identifying improved outcomes of care for patients treated by specialized and experienced teams at centers performing a high volume of surgical procedures,^4^, ^5^ particularly for more complex surgery such as pancreatic, esophageal, and prostate cancers. Prostate cancer is a relevant tumor type when considering the centralization of specialist services. First, the quality of the surgery has an impact on the chance of complete removal of the tumor whilst minimizing the risk of side effects, such as urinary incontinence and erectile dysfunction.^6^ Second, a volume outcome relationship has been established for different end‐points following prostate cancer surgery.^7^

In light of this, national cancer plans in the English National Health Service (NHS) have since 2002 advocated the centralization of specialist urological surgical services into fewer, high‐volume centers. The NHS provides treatment to more than 90% of cancer patients.^8^ A new geographical configuration was established, with local cancer units referring patients suitable for a radical prostatectomy to a regional specialist center.^9^, ^10^ However, a consequence of centralization is that it may require patients to travel further for treatment which could widen inequities in access for those less able to travel.^11^, ^12^

This is relevant considering a previous analysis for prostate cancer surgery which found that patterns of patient mobility has resulted in shifts in hospitals’ market shares.^13^ One in three men who had a radical prostatectomy for prostate cancer between 2010 and 2014 in the English NHS travelled beyond or bypassed their nearest prostate cancer surgery center. Men who were younger, fitter, and more affluent were more likely to travel to another surgery center than the one nearest to them.

This highlights concerns with respect to the increasing regionalization of specialist services. For example, increased travel times for cancer care could reduce treatment uptake for specific patient groups. This is particularly relevant for prostate cancer where competing radical treatment strategies exist (eg surgery, radiotherapy, and brachytherapy), which are often located at different geographic locations.^14^, ^15^ In addition, apparent advantages of the volume‐outcome relationship that may emerge from centralization of care may not be shared equally across the population, but instead concentrated in patients best able to access the benefit of centralization. Those patients are likely to be closer to high performing centers, or else younger, fitter, and more affluent.

### 1.1 Centralization of prostate cancer services in the NHS

Since 2010, 15 of 65 functioning prostate cancer surgery centers have been closed in the English NHS due to a combination of regional coordination between specialist centers as well as the effects of hospital competition and patient choice, partly driven by the adoption of robotic surgery.^16^ The integration of robotic surgery as a driver of centralization of prostate cancer surgery has been observed in several countries.^17^, ^18^, ^19^

The result of centralization on patient travel burden and treatment quality remains unknown, but it does highlight the need for a robust evidence‐based approach to health service planning. An important question is to what extent changes in the configuration of specialist services have a negative impact on the ability of all patients to access centralized specialist services which could lead to unintended consequences on patient outcomes.^20^

A recent study has used discrete choice experiments within small samples of patients, health professionals and the general public to get a better understanding of the acceptability of different centralization scenarios, focusing on the trade‐off between travel time and treatment outcomes for cancer surgery.^21^ The willingness to travel found by this study seems improbably large. It was estimated that people are willing to travel 75 minutes extra for a 1% lower absolute risk of complications and even more than 5 hours extra to reduce their risk of death by 1%. The extent to which these “stated” preferences can be used as a means of informing policy is debatable because responses to hypothetical scenarios and patients’ preferences “revealed” through their actual behavior may be different.^22^, ^23^, ^24^

There is an increasing body of literature that has attempted to simulate the effect of centralization of health care services based on parameters derived from population‐level administrative hospital data on actual patient visits. Previous studies attempting to assess the impact of centralization on travel times have followed the “distance minimization approach,” simply diverting patients who were treated at a center that is closed (as per the centralization scenario) to the *nearest* alternative center that would be still open after centralization.^11^, ^25^ An approach which only values distance or spatial access fails to acknowledge the significant number of other factors that account for patient preference for treatment center. Based on revealed preferences derived from studies of actual travel patterns, it is known that patients are prepared to travel beyond (bypass) their nearest hospital for treatment,^26^, ^27^ for example in response to the reputation of hospitals and their surgeons, the availability of innovative technologies, and waiting time.^28^

In this present study, we use data on actual travel patterns and an innovative simulation approach to provide a robust and comprehensive assessment of the impacts of three hypothetical re‐design scenarios on travel time and equity in access to radical prostatectomy services in the English NHS, a single‐payer system. Our simulation approach, however, can be applied by any authority, public or private, that is seeking to rationalize its health services into fewer centers nationally or regionally or within particular insurer catchment areas.

## 2 METHODS

### 2.1 Data sources and study population

For this study, we obtained individual patient‐level data on all men who were diagnosed with prostate cancer and underwent radical prostatectomy in the English NHS between January 1, 2010 and December 31, 2014 from the Hospital Episode Statistics (HES) database linked at the patient‐level to English cancer registry data. These data provide information on where patients actually received the treatment.

The population‐weighted centroids of the patients’ Lower Super Output Areas (geographic areas defined by the Office for National Statistics that typically includes 1500 residents or 650 households) and the full postcodes for the hospitals where the surgery was undertaken were inputted into a geographical information system (ESRI ArcGIS 10.3) to calculate travel times according to the fastest route by car (using Ordnance Survey MasterMap Integrated Transport Network). For each patient, the travel time to all prostate cancer surgical centers was calculated.

The HES dataset was used to determine patient‐level characteristics, including age, the number of comorbidities according to the Royal College of Surgeons Charlson comorbidity score,^29^ socioeconomic deprivation expressed in terms of quintiles of national distribution of the Index of Multiple Deprivation,^30^ hospital that provided the surgical treatment, and date of the surgical procedure. National cancer registry data were used as the data source for cancer stage, which was categorized according to a modified D’Amico classification system.^31^, ^32^

### 2.2 Centralization scenarios

For the purpose of our study, we created three pragmatic centralization scenarios to identify hypothetical closures of surgical units based on current clinical and policy discussion regarding quality improvement, patient experience, and efficient use of NHS resources.

In *Scenario A* (volume), surgical treatment for prostate cancer is restricted to centers undertaking more than 50 radical prostatectomies per annum. This scenario follows evidence supporting improvements in peri‐ and postoperative outcomes as well as function when surgery is performed in high‐volume relative to low‐volume units. The threshold of 50 procedures is based on current guidelines and the volume outcome literature.^7^

In *Scenario B* (facilities), surgical treatment is restricted to comprehensive cancer treatment centers. This is defined as centers having both surgical and radiotherapy facilities on site independent of volume. From the patient perspective, it is desirable when the major treatment options (eg surgery and radiotherapy) are available in the same center. Furthermore, comprehensive cancer centers provide all necessary support services for the management of patients (eg andrology services).

In *Scenario C* (capacity utilization), prostate cancer surgery is restricted to centers classified as “winners.” A previous analysis has demonstrated that patient choice has an impact on market share, creating “winners” and “losers” with some centers having a net gain of patients due to patient selection and others a net loss.^16^ Closing centers that have a net‐loss of patients to alternative centers could be considered to be a direct response to choices that patients seemed to have made themselves.

### 2.3 Centralization simulation analysis

Simulation analysis was conducted to assess the impact of centralization of prostate cancer surgery services on patient travel times. After simulating the closure of a number of cancer surgery centers, the probability of travelling to each of the remaining centers in the choice set was predicted for all individual patients, using a conditional logit choice model.^33^ Similar to other studies^26^ we restricted patient choice sets to reduce the computational burden as well as to avoid a potential bias caused by outliers. That is, choice sets included all prostate cancer surgery centers within 3 hours of travel time (which is about eight times patients’ median travel time for prostatectomy in the English NHS).

In addition to travel time from the patient's home to the prostate cancer surgery center, we included three center‐level characteristics: established robotic center, university teaching hospital, and strong media reputation. These were informed by a previous systematic review of the literature and qualitative interviews with both men previously treated for prostate cancer and uro‐oncology specialists currently practicing in the UK.^13^, ^28^, ^34^ A previous analysis has also demonstrated that men had greater odds of travelling to a center with one of these characteristics for prostate cancer surgery, independent of travel time, with one in three men bypassing their nearest center.^13^

We also included five patient characteristics: elderly (≥65 year), comorbidity, socioeconomic background and residence (residing in urban or rural area as well as residing in London or not). These characteristics were included as interaction terms with travel time in the conditional logit choice model.

Changes in travel burden resulting from centralization of surgery services were calculated as the difference between actual times travelled by patients for their radical prostatectomy precentralization and weighted average travel times postcentralization. The choice probabilities predicted by the conditional logit model were used as weights reflecting the relative importance of the remaining cancer centers to the patient. To compare this new simulation approach with the distance minimization approach used by previous simulation studies in this field of research, we also estimated the travel burden by assigning patients to their nearest available center postcentralization.

To study what the impact on travel time of closing cancer centers is according to patient characteristics, we estimated a multivariable linear regression model with change in travel burden as the dependent variable and the five patient characteristics as explanatory variables. In addition, pre‐ and postcentralization average travel times are presented graphically for different patient groups to illustrate increased inequities in treatment access. All analyses were undertaken in Stata version 14.

## 3 RESULTS

### 3.1 Patient sample and centralization scenarios

We studied 19,256 patients who were diagnosed with prostate cancer between January 2010 and December 2014, and who subsequently underwent a radical prostatectomy in the English NHS. We excluded 211 (1%) men who went to a cancer center more than 3 hours away from their home address as well as 16 men (0.1%) who had only one provider option within 3 hours.

**FIGURE 1:**
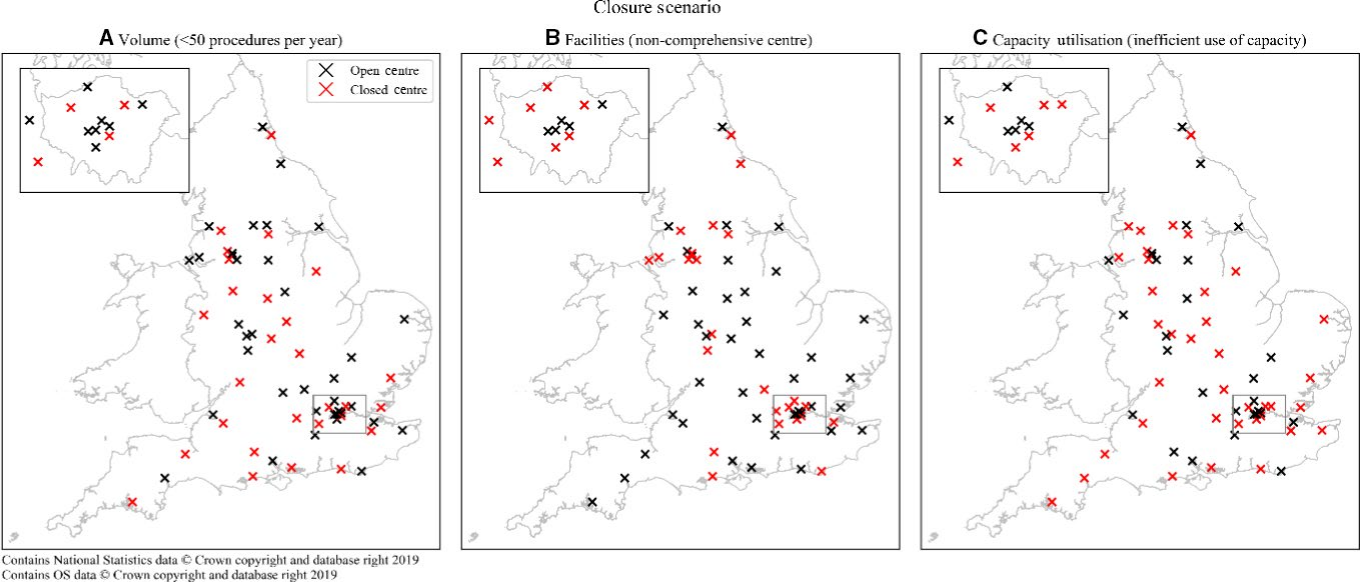
Location of open and closed prostate cancer surgery centres for each hypothetical centralisation scenario

The final sample was composed of 19,029 men living in England matched to 65 providers of prostate cancer surgery. Among the patients, 8046 (42%) were aged 65 and over, 1422 (7%) had at least one comorbidity, and 9064 (48%) lived in the most socio‐economically deprived areas (Table 1). In the sample, 4442 men (23%) lived in rural areas and 2656 (14%) in London. On average, patients travelled 31 minutes to their treatment center. For each scenario, the hypothetical closures of surgical units are represented in Figure 1. Table 1 presents the descriptive statistics of these patient subgroups as well as the total patient group.

**TABLE 1:** Sociodemographic characteristics of patients in centers that closed according to the centralization scenario

	Total patient cohort	Scenario A (volume)	Scenario B (facilities)	Scenario C (capacity utilization)
	65 centers	28 centers closing	24 centers closing	37 centers closing
	19,029 patients included	3993 patients moving to another center	5763 patients moving to another center	7896 patients moving to another center
	Number (%)	Number (%)	Number (%)	Number (%)
Aged 65 and over	8046 (42)	1689 (42)	2380 (41)	3302 (43)
Low socioeconomic status (national IMD quintiles 3‐5)	9064 (48)	1959 (49)	2797 (49)	3847 (50)
At least one comorbidity	1422 (7)	285 (7)	464 (8)	511 (7)
Place of residence				
Rural area	4442 (23)	1041 (26)	1021 (18)	1808 (24)
London	2656 (14)	247 (6)	637 (11)	778 (10)
Other urban area^a^	11,931(63)	2705 (68)	4105 (71)	5073 (66)

Note

Values are numbers with percentages in parentheses.

^a^ Residence in an urban area, but not in London.

The number of patients affected by the centralization differs across scenarios. Under scenario A (volume), in which 28 centers (43%) performing <50 prostatectomies per year are closed, 3993 men (21% of the total patient group between 2010 and 2014) would have to choose a new treatment location.

A total of 5763 men (30%) would be affected by centralization scenario B (facilities), in which prostate cancer surgical treatment is restricted to 41 comprehensive cancer treatment centers and 24 (37%) centers are closed.

Under scenario C (capacity utilization), in which 37 centers (57%) identified as having a net loss of patients are closed, 7896 men (41%) would reselect treatment location. Under this latter scenario, 237 men (3%) would have no alternative cancer center available within 3 hours of travel time. Their average travel time would increase from 50 to 213 minutes if they travelled to the nearest available center postcentralization. For this very small group of men we did not calculate weighted average travel times postcentralization because, as explained above, an underlying assumption of the estimated choice model is that patient choice sets only include cancer surgery centers within 3 hours of travel time.

### 3.2 Patient preferences

Table 2 reports the estimation results of the conditional logit choice model. Patients preferred to have surgery in a center requiring shorter travel time (odds ratio < 1). There are statistically significant differences in the impact of travel time between patient groups at the 1% level. The impact of travel time was greater in men from a socioeconomically more deprived area (national IMD quintiles 3‐5) than in men living in more affluent areas. This is demonstrated by the odds ratio of <1, indicating men from lower socioeconomic areas had a lower willingness to travel. The impact of travel time was also greater in men aged 65 and over, in men with comorbidity, and in men living in London and other urban areas. They had a lower willingness to travel (odds ratios < 1) than younger (<65), fitter (no comorbidities) men and those living in rural areas. Individuals also preferred to be treated in centers that provide robotic prostate cancer surgery and in centers that employ surgeons with a strong media reputation as demonstrated in a previous analysis.^13^

**TABLE 2:** Odds ratios (OR) from the conditional logit choice model estimating the probability of travelling to one of the prostate cancer surgery centers available within 3 h

	OR	95% confidence interval	*P*‐value
Travel time (in minutes) for base case patient^a^	0.920	0.918 to 0.922	<0.001
Interaction with patient characteristic^a^			
×Age ≥ 65	0.991	0.989 to 0.994	<0.001
×Low socioeconomic status (IMD score 3‐5)	0.996	0.994 to 0.999	0.003
×At least one comorbidity	0.987	0.981 to 0.992	<0.001
×London (compared to other Urban area)	0.846	0.837 to 0.854	<0.001
×Rural area (compared to other Urban area)	1.021	1.018 to 1.023	<0.001
Strong media reputation	1.933	1.841 to 2.028	<0.001
University‐teaching hospital	0.928	0.889 to 0.970	0.001
Established robotic center	1.756	1.655 to 1.862	<0.001
N observations	505,045		
N patients	19,029		

^a^ The base case patient represents an individual with the following characteristics: Age < 65, socioeconomic status—high (IMD 1‐2), No comorbidities, living in an Urban area (not London). The impact of the patient characteristics on travel time is presented as interaction terms. These should be multiplied with the adjusted OR for “travel time” for the base case patient (0.920) to formulate a new OR. Interaction terms can be used in any combination to assess the effect of different patient characteristics on the odds that a patient travels to a particular hospital. As an example, to calculate the new OR for an elderly man (age ≥ 65), with at least one comorbidity, living in London, but still of high socioeconomic status—multiply 0.920 by the corresponding interaction term for men who are elderly (0.991), have comorbidity (0.987) and who live in London (0.846). The new odds ratio is 0.920 × 0.991 × 0.987 × 0.846 = 0.761. Men with this sociodemographic profile have a lower willingness to travel than the base case patient described.

### 3.3 Impact of centralization on travel time

**FIGURE 2:**
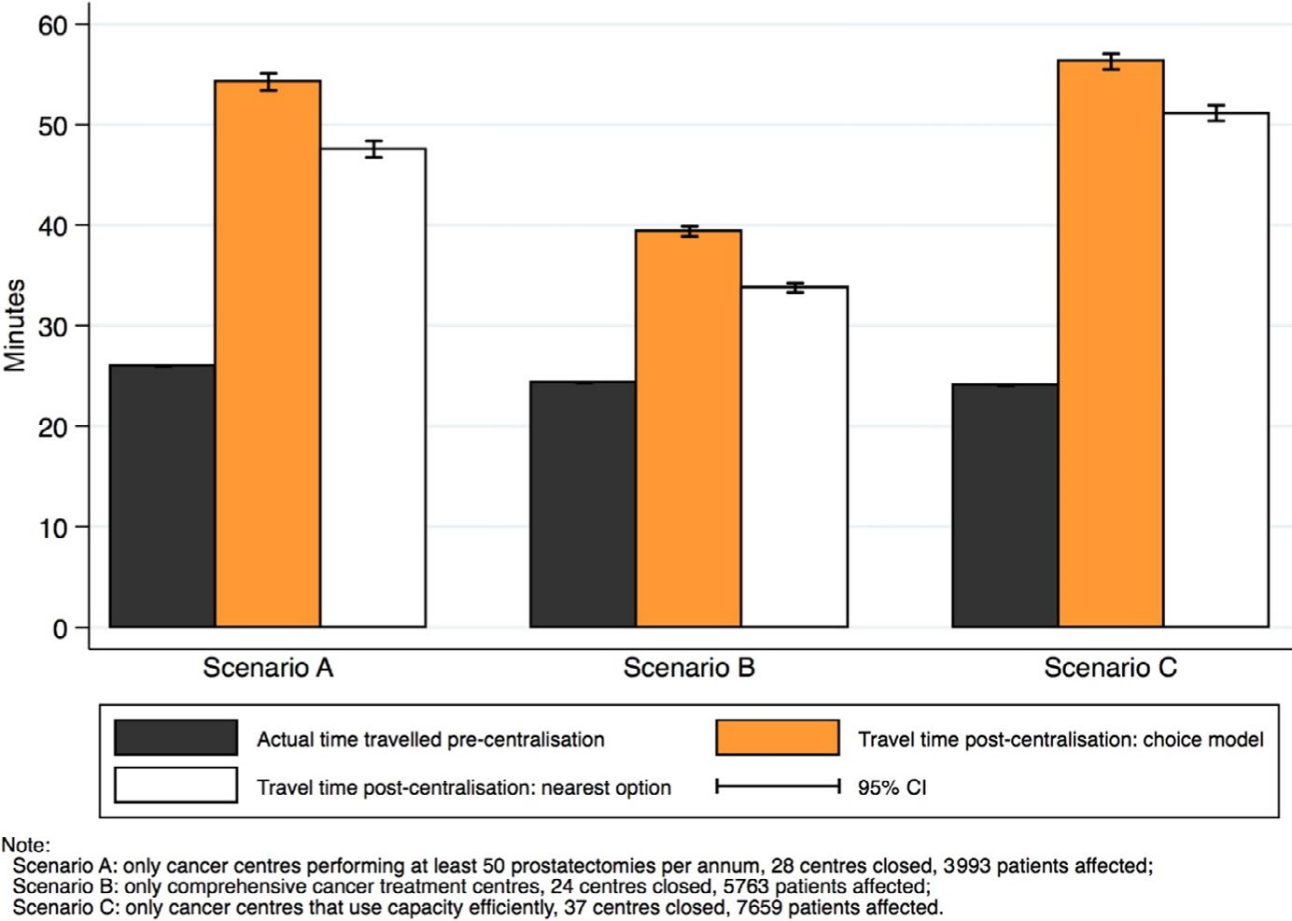
Average time travelled pre‐centralisation and average travel time expected postcentralisation in minutes for scenarios A (volume), B (facilities), and C (capacity utilisation)

For each centralization scenario there is a substantial increase in the expected patient travel time using the estimated conditional logit choice model (Figure 2, orange bars). Patients affected by the centralization in scenario A had to travel an additional 28 minutes on average with postcentralization average travel time approaching 1 hour. Hence, travel time would more than double (108% relative increase) on average for them. Under centralization scenario B the additional travel time for the affected patients would be on average 15 minutes (+63%). Finally, in centralization scenario C, patients reselecting their treatment location would be expected to travel an additional 32 minutes (+133%).

Figure 2 also considers what happens if patients are diverted to their next nearest center (distance minimization approach). As expected, the increase in average patient travel time for those affected by centralization is consistently less with this approach compared to the simulation approach which considers patients’ preferences. For example, patients would have to travel 22 more minutes (+85%) on average under scenario A if they would go to the next nearest center compared to 28 more minutes if they would travel to a center according to the predictions of the conditional logit choice model.

### 3.4 Impact of centralization on inequities in access

Table 3 demonstrates the results of the multivariable linear regression analysis examining the average impact of each centralization scenario on specific patient groups’ travel time. In each scenario, as shown by the results for the base case patient, there is a significant increase in patient travel burden ranging from 16 minutes in scenario B to 30 minutes in scenario C.

**TABLE 3:** Impact of different centralization scenarios on travel time according to patient characteristics. Results of multivariable regression

	Scenario A (volume)		Scenario B (facilities)		Scenario C (capacity utilization)	
	3993 patients		5763 patients		7659 patients	
Increase in travel time (95% CI) (min)						
Increase in travel time for base case patient^a^	29.10 (27.75 to 30.45)	*P* < 0.001	16.46 (15.44 to 17.49)	*P* < 0.001	30.19 (29.10 to 31.28)	*P* < 0.001
Difference in increase in travel time compared to base case patient						
Age ≥ 65	−0.74 (−2.23 to 0.76)	*P* = 0.334	−1.46 (−2.60 to − 0.33)	*P* = 0.012	−0.05 (−1.22 to 1.13)	*P* = 0.940
Low socioeconomic status (IMD score 3‐5)	−0.80 (−2.30 to 0.69)	*P* = 0.292	1.32 (0.17 to 2.46)	*P* = 0.024	1.70 (0.52 to 2.87)	*P* = 0.005
At least one comorbidity	2.86 (0.00 to 5.73)	*P* = 0.050	−1.10 (−3.16 to 0.95)	*P* = 0.293	−0.73 (−3.06 to 1.60)	*P* = 0.538
London (compared to other Urban area)	−23.25 (−26.36 to − 20.13)	*P* < 0.001	−12.96 (−14.78 to − 11.14)	*P* < 0.001	−22.69 (−24.66 to − 20.73)	*P* < 0.001
Rural (compared to other Urban area)	4.31 (2.62 to 6.01)	*P* < 0.001	0.47 (−1.03 to 1.97)	*P* = 0.539	15.08 (13.68 to 16.47)	*P* < 0.001

^a^ The base case patient represents an individual with the following characteristics: Age < 65, socioeconomic status—high (IMD 1‐2), no comorbidities, living in an Urban area (not London).

For patients from London the increase in travel time is much less than for patients living in a rural area. For example, under scenario C the average increase in patient travel time for London dwellers is 23 minutes less, all else being equal.

Under scenarios A and C, for patients living in rural areas the increase in travel time is higher than for patients living in urban areas. The biggest impact is noted in scenario C (+15 minutes compared to patients from urban areas).

All three centralization scenarios would result in a very small decrease in travel times for older patients (>65 years old) compared to their younger counterparts, although this was only statistically significant for scenario B (average adjusted difference of −1.5 minutes). For patients with at least one recorded comorbidity, scenario A results in a significantly higher but very marginal increase in travel time (average adjusted difference +2.8 minutes) compared to patients with no comorbidity. For patients from less affluent areas (IMD score 3‐5), both scenario B and C result in a significantly higher but very small increase in travel time (average adjusted differences of +1.3 and +1.7 minutes, respectively) compared to those living in more affluent areas.

The model enables an evaluation of men with different patient characteristics (Table 3). For example, under scenario C a man of lower socioeconomic status living in a rural area would have to travel an additional 46.7 minutes (30.19 + 15.08 + 1.70) compared to an additional 13.4 minutes (30.19 − 1.70) for an affluent man living in an urban area.

Appendices S1‐S3 present the variation in impact of the three centralization scenarios across different patient groups, based on combinations of different characteristics that have been found to be statistically significant from the multivariable regression analyses as presented in Table 3.

## 4 DISCUSSION

This study provides an innovative simulation approach for assessing the impact of centralizing complex cancer surgery services on patients’ travel burden. Using individual patient‐level data on all men who underwent radical prostatectomy in the English NHS between 2010 and 2014, we considered three pragmatic scenarios for centralization of prostate cancer surgery services: scenario A (volume), scenario B (facilities), and scenario C (capacity utilization).

Compared to previous studies in this field of research, our approach explicitly takes into account patients’ preferences revealed through their actual hospital selections by using data on patient visits from administrative patient datasets to model patient choice. Travel times postcentralization are calculated as a weighted average of travel times to remaining cancer centers using the probabilities predicted by an estimated conditional logit choice model as weights. Our approach therefore results in more realistic predictions than previous studies that simply assumed that patients affected by centralization would go to their nearest alternative center that would be still open.

For each of the centralization scenarios an overall increase in average travel burden is apparent, with the smallest impact found for scenario B (+15 minutes) and the biggest impact found for scenarios A (+28 minutes) and C (+32 minutes). Different scenarios have a different overall impact on (average) travel time and therefore on equity given the reduced willingness to travel of older, sicker, and lower socioeconomic groups. Of note, particularly under scenario C, extra travel time substantially differs according to whether patients live in rural or an urban area.

The results provide more general insights into the implications of a program of surgical service centralization on predicted travel burden, equity, and efficiency of the service. First, the use of a pure distance minimization approach in understanding travel burden on patients would not fully capture the impact of a patient's own personal characteristics and personal preferences for particular hospital characteristics. This is relevant in the context of the NHS and other health systems supporting patient choice.^28^

Second, the use of national rather than regional datasets provides a clear understanding on the differential impact of national top‐down policies on travel burden. Specifically, uniform policy criteria, disproportionately affect patients living in rural areas, who on average have to travel significantly further compared to those living in urban areas, which demonstrates the difficulties of “one size fits all” centralization policies. This is most clearly observed in Scenario C with its increases in travel time especially for patients in rural areas. This may result in lower utilization of curative and palliative treatments, creating regional inequities in outcomes.

Conversely, one can see how such mechanisms for service re‐design may have a negligible impact on travel burden for patients in highly urbanized regions such as London. In these settings, the capacity of centers to expand their service would be the next criteria to consider. One caveat with our current approach is the use of private drive times alone as public transport times were not available. This could impact on the observed differences and behaviors, in the different regions.

Third, in addition to the overall increase in travel time which is likely to be more problematic for vulnerable patient groups, it was noted that patients’ personal characteristics (eg socioeconomic status) could affect equity in access gradients further. All three centralization scenarios result in an increase in travel time for patients, however, the extra travel burden on specific patient groups resulting from these centralization scenarios is in reality very small.

Fourth, scenarios A and B result in similar numbers of cancer surgical unit closures (28 and 24 respectively), but very different impacts on patient travel burden that can inform policy. The findings suggest that efficiencies could be achieved by closing noncomprehensive cancer centers (scenario B), as the expected average increase in travel burden of 15 minutes is almost half that expected from Scenario A (28 minutes). The closure of centers in scenario B could result in increases in the number of procedures performed at the remaining 41 centers assuming demand remains the same, and hence the objectives for A (creating high volume radical prostatectomy centers) and B (ensuring each cancer center is a comprehensive cancer center) may be achievable through a single policy.

Scenario C, considers a different scenario, whereby centers, that are “losing” patients to other centers should be closed given preferences for alternative centers. Hence, patients through their choices and that of their primary care physicians, can influence which services remain open. However, the impact on patients with respect to travel burden is significant, particularly for rural dwellers, who face additional travel times of up to 45 minutes. The map in Figure 1C, shows that surgical units expected to close under this scenario would cover substantial catchment areas (eg Cornwall, Devon, Norfolk, Kent, Cumbria) where few alternative centers are available and patients experience a significant travel burden in accessing local services. In addition, this could have a detrimental impact on access to specialist care. It also informs us that, within the NHS, a capacity maximization approach is unlikely to be achievable given the need to ensure equitable access nationally. Hence, centers may continue to operate despite having surplus capacity to protect access.

Although the simulation approach presented in this paper used data from a single‐payer health system, it is certainly adaptable across different contexts. Our study shows how the impact of centralization options can be empirically investigated if relevant patient data are available. Our methodological approach can be further developed to incorporate information on hospital quality and patient outcomes; for example, rates of toxicity at the provider‐level for patients undergoing radical prostatectomy.^35^ One can then model the effect of changes in provider‐level quality on willingness to travel, and how this varies between different patient groups as previously described.^36^ With respect to travel burden, it is not just travel time or distance that needs to be considered but also its cost to the patient. In addition, where provision of surgery and radiotherapy is not at a single site, the differential impact of the closure of such a site on the uptake of each of these treatment modalities can be considered.^37^

Further simulations could estimate the expected improvements or worsening of patient outcomes that may result from centralization. In this way we can observe directly the trade‐offs between travel times, equity, and quality, which need to be considered with health service planning.^25^ This modeling approach could also be used to fit the *k* best centers to close, such that differences between population groups and/or headline travel times are minimized. This would present an a‐theoretic, data driven comparator to the top‐down policy approach simulated here.

## 5 CONCLUSION

In this paper, we present an innovative simulation approach using national patient‐level datasets to understand better the impact of service re‐design on patient travel burden and equity in access to services. Our study results show how in the English NHS the additional travel burden associated with unit closures is regionally patterned and can widen inequities in access for particular patient groups, particularly those living in rural areas. Equally it also demonstrates, how in certain scenarios, quite significant centralization of the service, with closure of just over one‐third of current centers (24 of 65 centers), may result in only a relative small impact on patients with respect to travel time. Future work should focus on better understanding the trade‐offs between equity, travel burden, and patient outcomes to inform health care services re‐design. In this regard, we expect the model to be applicable to other tumor types and specialist disciplines.

## CONFLICT OF INTEREST

The authors declare no conflicts of interest.

## AUTHOR CONTRIBUTIONS

Conceptualization (AA, SvdG, MV), data curation (AA, DL) formal analysis (SvdG, AA, DL,), funding acquisition (N/A), methodology (AA, SvdG, DL, MV), project administration (AA), supervision (MV, JvdM), writing—original draft (AA, SvdG), and writing ‐ review and editing (All authors). AA and SvdG are joint first authors.

## Supporting information

Appendix S1‐S3

## Data Availability

The data that support the findings of this study are available from Public Health England. Restrictions apply to the availability of these data, which were used under license for this study.

## References

[1] Wyld L , Audisio RA , Poston GJ . The evolution of cancer surgery and future perspectives. Nat Rev Clin Oncol. 2015;12(2):115‐124. 25384943 10.1038/nrclinonc.2014.191

[2] Morche J , Renner D , Pietsch B , et al. International comparison of minimum volume standards for hospitals. Health Policy. 2018;122(11):1165‐1176. 30193981 10.1016/j.healthpol.2018.08.016

[3] Urbach DR . Pledging to eliminate low‐volume surgery. N Engl J Med. 2015;373(15):1388‐1390. 26444728 10.1056/NEJMp1508472

[4] Birkmeyer JD , Siewers AE , Finlayson EVA , et al. Hospital volume and surgical mortality in the United States. N Engl J Med. 2002;346(15):1128‐1137. 11948273 10.1056/NEJMsa012337

[5] Gruen RL , Pitt V , Green S , Parkhill A , Campbell D , Jolley D . The effect of provider case volume on cancer mortality: systematic review and meta‐analysis. CA Cancer J Clin. 2009;59(3):192‐211. 19414631 10.3322/caac.20018

[6] Vickers AJ , Savage CJ , Hruza M , et al. The surgical learning curve for laparoscopic radical prostatectomy: a retrospective cohort study. Lancet Oncol. 2009;10(5):475‐480. 19342300 10.1016/S1470-2045(09)70079-8 PMC2777762

[7] Trinh Q‐D , Bjartell A , Freedland SJ , et al. A systematic review of the volume‐outcome relationship for radical prostatectomy. Eur Urol. 2013;64(5):786‐798. 23664423 10.1016/j.eururo.2013.04.012 PMC4109273

[8] The King's Fund . The UK Private Health Market. London: King's Fund; 2014.

[9] National Institute for Health and Care Excellence . Improving outcomes in Urological Cancers – Guidance on Cancer Services. London: National Institute for Health and Care Excellence; 2002.

[10] NHS England . National Cancer Peer Review Programme: Manual for Cancer Services: Urology Measures. London: NHS England; 2014.

[11] Kobayashi D , Otsubo T , Imanaka Y . The effect of centralization of health care services on travel time and its equality. Health Policy. 2015;119(3):298‐306. 25480458 10.1016/j.healthpol.2014.11.008

[12] Versteeg SE , Ho VKY , Siesling S , Varkevisser M . Centralisation of cancer surgery and the impact on patients' travel burden. Health Policy. 2018;122(9):1028‐1034. 30060899 10.1016/j.healthpol.2018.07.002

[13] Aggarwal A , Lewis D , Charman SC , et al. Determinants of patient mobility for prostate cancer surgery: a population‐based study of choice and competition. Eur Urol. 2018;73(6):822‐825. 28760646 10.1016/j.eururo.2017.07.013

[14] Parry MG , Sujenthiran A , Cowling TE , et al. Impact of cancer service centralisation on the radical treatment of men with high‐risk and locally advanced prostate cancer: a national cross‐sectional analysis in England. Inte J Cancer. 2019;145(1):40‐48. 10.1002/ijc.32068 PMC6590431 30549266

[15] Aggarwal A , Nossiter J , Cathcart P , et al. Organisation of prostate cancer services in the English National Health Service. Clin Oncol. 2016;28(8):482‐489. 10.1016/j.clon.2016.02.004 26947316

[16] Aggarwal A , Lewis D , Mason M , Purushotham A , Sullivan R , van der Meulen J . Effect of patient choice and hospital competition on service configuration and technology adoption within cancer surgery: a national, population‐based study. Lancet Oncol. 2017;18(11):1445‐1453. 28986012 10.1016/S1470-2045(17)30572-7 PMC5666166

[17] Groeben C , Koch R , Baunacke M , Wirth MP , Huber J . High volume is the key for improving in‐hospital outcomes after radical prostatectomy: a total population analysis in Germany from 2006 to 2013. World J Urol. 2017;35(7):1045‐1053. 27933389 10.1007/s00345-016-1982-4

[18] Riikonen J , Kaipia A , Petas A , et al. Initiation of robot‐assisted radical prostatectomies in Finland: impact on centralization and quality of care. Scand J Urol. 2016;50(3):149‐154. 26881411 10.3109/21681805.2016.1142471

[19] Stitzenberg KB , Wong YN , Nielsen ME , Egleston BL , Uzzo RG . Trends in radical prostatectomy: centralization, robotics, and access to urologic cancer care. Cancer. 2012;118(1):54‐62. 21717436 10.1002/cncr.26274 PMC3184375

[20] Stitzenberg KB , Sigurdson ER , Egleston BL , Starkey RB , Meropol NJ . Centralization of cancer surgery: implications for patient access to optimal care. J Clin Oncol. 2009;27(28):4671‐4678. 19720926 10.1200/JCO.2008.20.1715 PMC3039919

[21] Vallejo‐Torres L , Melnychuk M , Vindrola‐Padros C , et al. Discrete‐choice experiment to analyse preferences for centralizing specialist cancer surgery services. Br J Surg. 2018;105(5):587‐596. 29512137 10.1002/bjs.10761 PMC5900867

[22] Wardman M . A comparison of revealed preference and stated preference models of travel behaviour. J Transp Econ Policy. 1988;22(1):71‐91.

[23] Harrison M , Milbers K , Hudson M , Bansback N . Do patients and health care providers have discordant preferences about which aspects of treatments matter most? Evidence from a systematic review of discrete choice experiments. BMJ Open. 2017;7(5):e014719. 10.1136/bmjopen-2016-014719 PMC5623426 28515194

[24] Quaife M , Terris‐Prestholt F , Di Tanna GL , Vickerman P . How well do discrete choice experiments predict health choices? A systematic review and meta‐analysis of external validity. Eur J Health Econ. 2018;19(8):1053‐1066. 29380229 10.1007/s10198-018-0954-6

[25] Poeran J , Borsboom GJJM , de Graaf JP , et al. Does centralisation of acute obstetric care reduce intrapartum and first‐week mortality? An empirical study of over 1 million births in the Netherlands. Health Policy. 2014;117(1):28‐38. 24703856 10.1016/j.healthpol.2014.03.009

[26] Varkevisser M , Van Der Geest SA . Why do patients bypass the nearest hospital? An empirical analysis for orthopaedic care and neurosurgery in the Netherlands. Eur J Health Econ. 2007;8(3):287‐295. 17256180 10.1007/s10198-006-0035-0

[27] Gutacker N , Siciliani L , Moscelli G , Gravelle H . Choice of hospital: which type of quality matters? J Health Econ. 2016;50:230‐246. 27590088 10.1016/j.jhealeco.2016.08.001 PMC5138156

[28] Aggarwal A , Lewis D , Mason M , Sullivan R , van der Meulen J . Patient mobility for elective secondary health care services in response to patient choice policies: a systematic review. Med Care Res Rev. 2016;74(4):379‐403. 27357394 10.1177/1077558716654631 PMC5502904

[29] Armitage JN , van der Meulen JH , Royal College of Surgeons Co‐morbidity Consensus G . Identifying co‐morbidity in surgical patients using administrative data with the Royal College of Surgeons Charlson Score. Brit J Surg. 2010;97(5):772‐781. 20306528 10.1002/bjs.6930

[30] Department of Communities and Local Government . The English Indices of Deprivation 2010. London: Department of Communities and Local Government; 2011.

[31] D'Amico AV , Whittington R , Malkowicz SB , et al. Biochemical outcome after radical prostatectomy, external beam radiation therapy, or interstitial radiation therapy for clinically localized prostate cancer. JAMA. 1998;280(11):969‐974. 9749478 10.1001/jama.280.11.969

[32] Royal College of Surgeons of England . National Prostate Cancer Audit ‐ First Year Annual Report – Organisation of Services and Analysis of Existing Clinical Data. London: The Royal College of Surgeons of England; 2014.

[33] McFadden D . Conditional Logit Analysis of Qualitative Choice Behavior. Zarembka P, ed. New York: Academic Press; 1973.

[34] Aggarwal A , Bernays S , Payne H , van der Meulen J , Davis C . Hospital choice in cancer care: a qualitative study. Clin Oncol. 2018;30(7):e67‐e73. 10.1016/j.clon.2018.03.009 29680734

[35] Sujenthiran A , Charman SC , Parry M , et al. Quantifying severe urinary complications after radical prostatectomy: the development and validation of a surgical performance indicator using hospital administrative data. BJU Int. 2017;120(2):219‐225. 28075516 10.1111/bju.13770

[36] Varkevisser M , van der Geest SA , Schut FT . Assessing hospital competition when prices don't matter to patients: the use of time‐elasticities. Int J Health Care Finance Econ. 2010;10(1):43‐60. 19662527 10.1007/s10754-009-9070-6

[37] Hamdy FC , Donovan JL , Lane JA , et al. 10‐Year outcomes after monitoring, surgery, or radiotherapy for localized prostate cancer. N Engl J Med. 2016;375(15):1415‐1424. 27626136 10.1056/NEJMoa1606220

