# Supplementary material for: Simulating the impact of centralization of prostate cancer surgery services on travel burden and equity in the English National Health Service: A national population based model for health service re‐design
Source: Cancer Med. 2020 Apr 23;9(12):4175–84. doi: 10.1002/cam4.3073 (PMC7300407; doi:10.1002/cam4.3073)
Supplement: Supplementary file 1 — Appendix S1‐S3 [file CAM4-9-4175-s001.docx]

**APPENDICES**

**Appendix 1 – Pre- and post-scenario A average travel times for different patient subgroups**

**
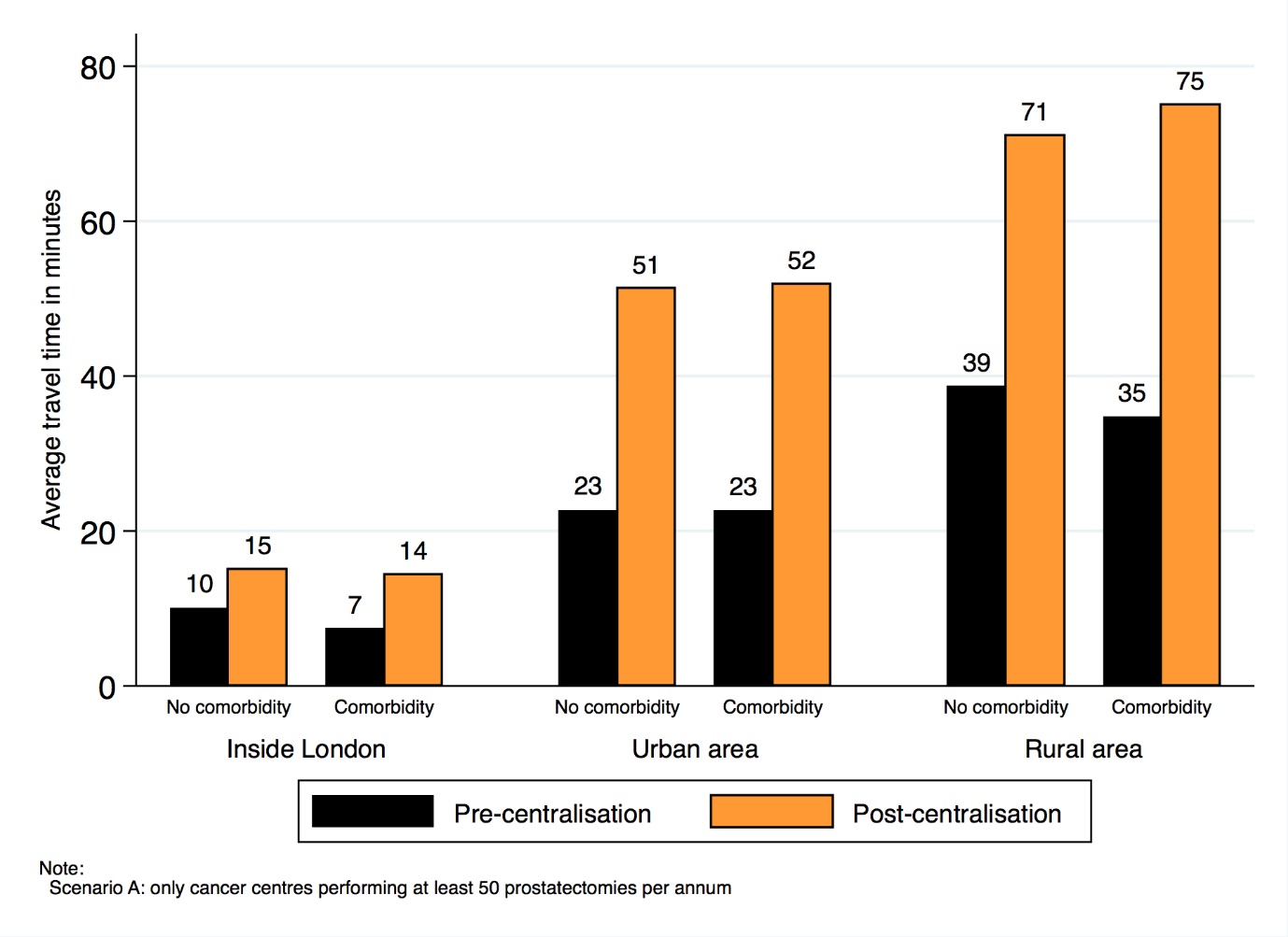
**

**Appendix 2 – Pre- and post-scenario B average travel times for different patient subgroups**

**
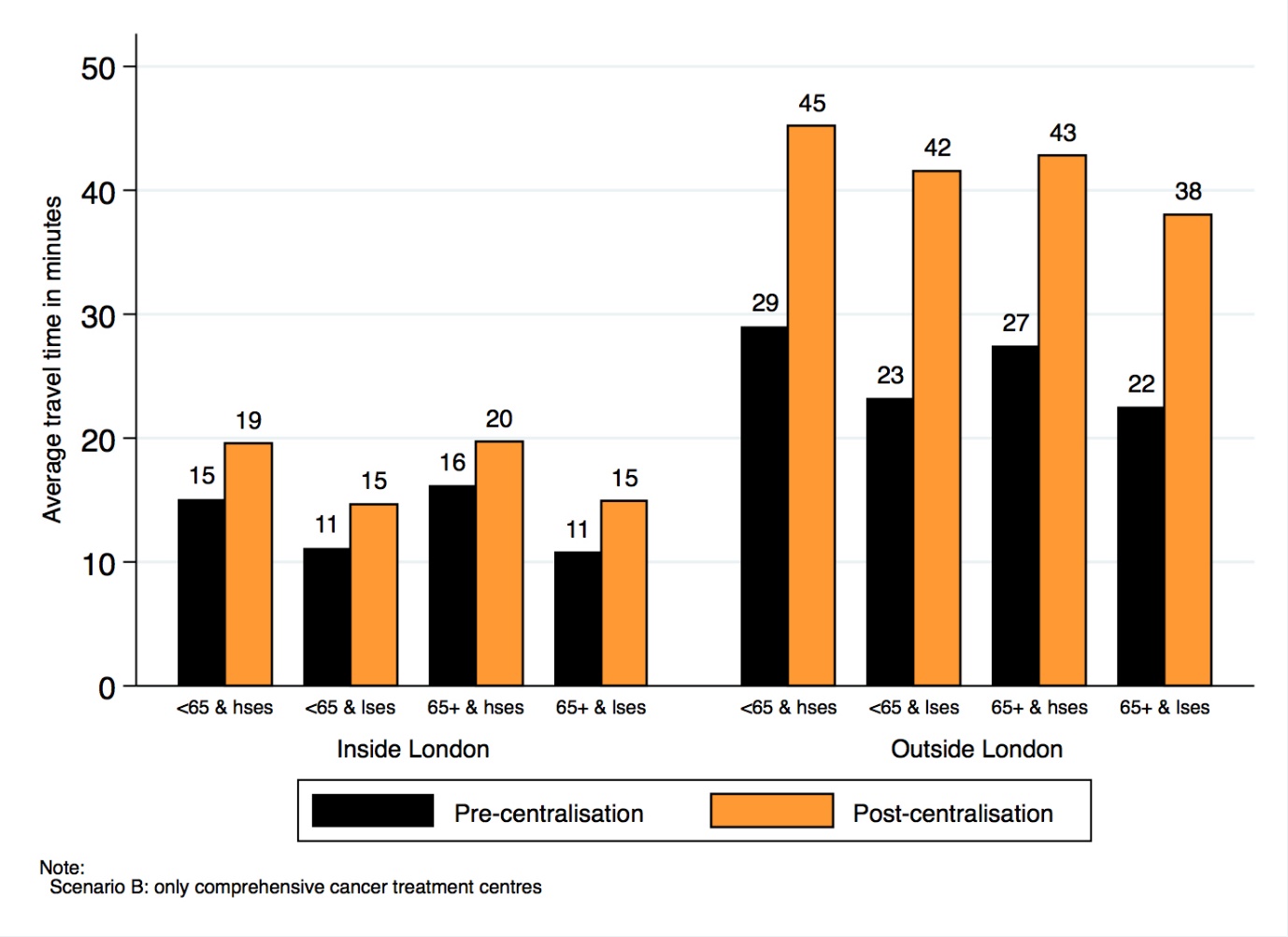
**

**Appendix 3 – Pre- and post-scenario C average travel times for different patient subgroups**

**
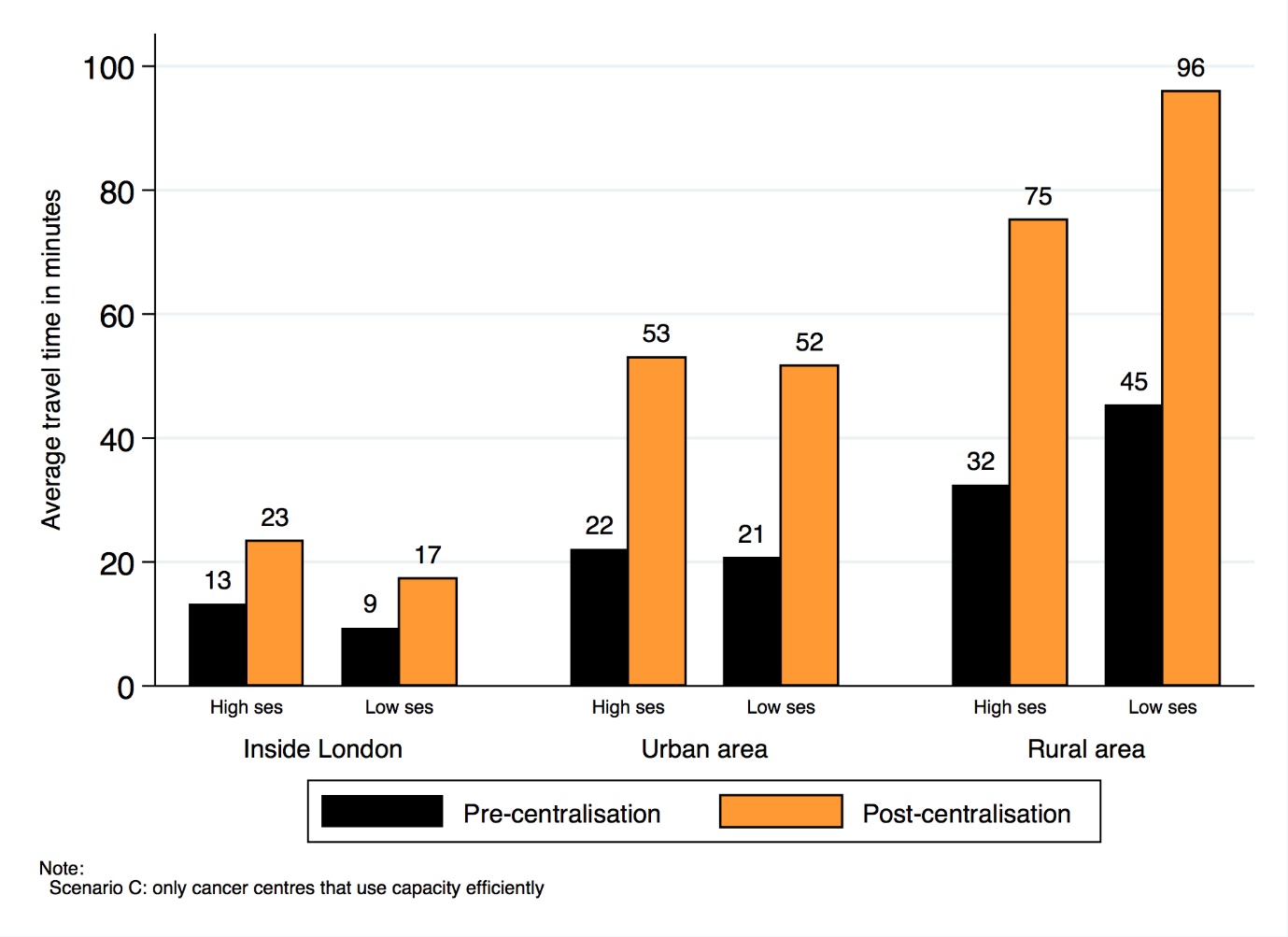
**
